# Distribution Characteristics of Antimicrobial-Resistance and Virulence-Associated Genes of *Bacillus cereus* in Animal-Origin Food in Guangzhou, China

**DOI:** 10.3390/microorganisms14092116

**Published:** 2026-09-21

**Authors:** Weiqi Liu, Wanxin Lin, Zhengyuan He, Peng Wan, Juntao Li, Wenguang Xiong, Zhenling Zeng

**Affiliations:** 1 Guangdong Provincial Key Laboratory of Veterinary Pharmaceutics Development and Safety Evaluation, South China Agricultural University, Guangzhou 510642, China; 2 National Risk Assessment Laboratory for Antimicrobial Resistance of Animal Original Bacteria, South China Agricultural University, Guangzhou 510642, China; 3 National Laboratory of Safety Evaluation (Environmental Assessment) of Veterinary Drugs, South China Agricultural University, Guangzhou 510642, China; 4 National Reference Laboratory of Veterinary Drug Residues, South China Agricultural University, Guangzhou 510642, China

**Keywords:** *Bacillus cereus* group, smoked sausage, animal-origin food, antimicrobial-resistance genes, virulence-associated genes, biofilm formation

## Abstract

This study investigated the prevalence and genomic characteristics of Bacillus cereus group organisms in animal-origin foods sold in Guangzhou, China. A total of 505 retail samples, including 117 milk samples and 388 commercially available smoked sausage products, were collected from supermarkets and farmers’ markets. The isolation rates of *B. cereus* group organisms were 8.5% in milk and 44.9% in smoked sausages. Whole-genome sequencing of 169 isolates showed that antimicrobial-resistance genes, including bacII, fosB and vanZF, and diarrheal virulence-associated genes, including nheA, nheB and nheC, were widely distributed among the isolates. Most representative isolates showed biofilm-forming ability, and three antimicrobial-resistant isolates carrying multiple virulence-associated genes showed pathogenic potential in mice under high-dose experimental administration. These findings indicate that retail smoked sausage products can serve as a reservoir of *B. cereus* group organisms with antimicrobial-resistance, virulence-associated and biofilm-forming traits. However, the detection of resistance- or virulence-associated genes does not necessarily prove gene expression, toxin production, phenotypic resistance or consumer exposure risk. This study did not directly evaluate growth of *B. cereus* in sausage matrices during storage. Therefore, the present results indicate a potential microbiological hazard rather than a quantified consumer risk. Product-specific challenge studies under defined storage conditions are required to determine whether *B. cereus* can proliferate in particular sausage products and reach hazardous levels at consumption.

## 1. Introduction

*Bacillus cereus* is a Gram-positive, spore-forming opportunistic foodborne pathogen that can cause emetic and diarrheal syndromes and, in susceptible individuals, severe extraintestinal infections [1]. The pathogenic potential of *B. cereus* group organisms is associated with multiple virulence-associated determinants, including enterotoxin genes, emetic toxin gene clusters, enzymes, motility-related traits and biofilm-forming capacity. With the extensive use of antimicrobial agents, antimicrobial-resistance-associated determinants in *B. cereus* group organisms have also attracted increasing attention because they may complicate treatment and facilitate dissemination through food-production environments.

*B. cereus* group organisms are widely distributed in soil, water, food-processing environments and foods of animal or plant origin. In the food industry, their ability to form spores and biofilms facilitates persistence on processing equipment and may cause secondary contamination during production, storage and retail handling. Biofilms formed on stainless steel, rubber and plastic surfaces can protect internal cells from disinfectants and may continuously release spores or vegetative cells into food-production chains.

Previous surveillance studies have reported the occurrence of *B. cereus* group organisms in multiple food categories, including dairy products, cereal-based foods and meat products [2,3,4,5]. However, the detection of *B. cereus* group organisms in foods should be distinguished from the confirmation of growth in a specific matrix and from quantitative consumer-risk assessment. Actual risk at consumption depends on initial contamination, bacterial growth, toxin production, storage temperature, pH, water activity, salt concentration, packaging conditions and consumption pattern.

According to reports from the European Food Safety Authority (EFSA) and the Centers for Disease Control and Prevention (CDC), foodborne disease outbreaks caused by *B. cereus* consistently rank among the most frequent worldwide. The contamination issue is particularly prominent in Asian regions with staple cereal-based diets. Previous studies have indicated that the detection rate of *B. cereus* in retail rice products, ready-to-eat fruits and vegetables, dairy products, and spices remains steadily between 20% and 50% [3]. Especially for Chinese ready-to-eat foods, improper storage temperatures after cooking (e.g., long-term warm preservation) enable residual spores to germinate and proliferate rapidly, easily exceeding the often-cited reference threshold of 10^5^ CFU/g.

Therefore, systematic investigation of the prevalence, antimicrobial-resistance gene profiles, virulence-associated gene profiles and biofilm-forming ability of *B. cereus* group isolates from animal-origin foods is important for food-safety surveillance. In addition, it is necessary to distinguish microbiological hazard identification from actual consumer risk assessment. The presence of *B. cereus* group organisms or virulence-associated genes indicates potential risk, whereas confirmation of growth in a food matrix, toxin production and hazardous exposure levels require product-specific challenge studies. Accordingly, this study aimed to (i) determine the isolation rates of *B. cereus* group organisms from retail milk and commercially available smoked sausage products in Guangzhou; (ii) characterize antimicrobial-resistance and virulence-associated gene distributions by whole-genome analysis; (iii) evaluate biofilm-forming ability of representative isolates; and (iv) assess the pathogenic potential of selected antimicrobial-resistant and virulence-associated isolates in a mouse model.

## 2. Materials and Methods

### 2.1. Sample Source, Product Description and Isolation of B. cereus Group Organisms

A total of 505 retail animal-origin food samples were collected from supermarkets and farmers’ markets in Guangzhou, Guangdong Province, China, including 117 milk samples and 388 commercially available smoked sausage products. The sausage samples were market-available smoked products made from fresh raw meat before smoking/processing; they were analyzed as purchased retail products rather than raw meat. Because the products were not subjected to independent process verification in this study, we describe them as commercially available smoked sausage products instead of assuming a universal fully cooked or ready-to-eat status. The quality-control strains used in this study were *B. cereus* CMCC(B)63303 and Staphylococcus aureus ATCC 29213. Isolation and detection of *B. cereus* group organisms were performed according to the Chinese National Food Safety Standard GB 4789.14-2014 [6] (Food Microbiological Examination: Examination of *Bacillus cereus*). Briefly, each sample was aseptically homogenized, enriched or diluted according to the standard procedure, plated on selective medium for *B. cereus* group detection, incubated under the recommended conditions, and presumptive colonies with typical morphology were purified and confirmed by standard biochemical identification and/or genome-based identification. All procedures were performed under the same standardized workflow to ensure comparability among samples.

### 2.2. Whole-Genome Sequencing and Genomic Annotation

Genomic DNA was extracted from overnight bacterial cultures using the Bacterial DNA Kit (Tiangen, Beijing, China) following the manufacturer’s instructions. DNA concentration and quality were assessed with a Qubit Fluorometer (ThermoFisher Scientific, Waltham, MA, USA) using the Qubit dsDNA HS Assay. Sequencing libraries were constructed with the Hieff NGS^®^ MaxUp II DNA Library Prep Kit for Illumina (Yeasen, Shanghai, China). Paired-end sequencing (2 × 250 bp reads) was performed on the Illumina MiSeq platform by Shenggong Bio (Shanghai, China). Raw sequencing reads were quality-trimmed using Sickle v1.33, and read quality was evaluated with FastQC v3.17.0. De novo genome assembly was conducted in CLC Genomics Workbench (Version 10.0.1, CLC Bio, Aarhus, Denmark).

Assembled contigs were further filtered for genome quality using QUAST v5.0.2 and CheckM v1.2.1. Only assemblies with completeness > 97.5%, contamination < 2.5%, contig number < 1000, and N50 > 20 kbp were retained for downstream analysis. Species-level taxonomic assignment for Bacillus cereus group isolates was performed using FastANI v1.3 and GTDB-Tk v1.3.0. BTyper v2.3.3 was applied for panC group assignment, rpoB allelic typing and MLST profiling.

Detection of virulence-associated genes was implemented in BTyper v2.3.3 with default cut-offs: minimum amino acid identity of 50% and minimum query coverage of 70%. Antimicrobial-resistance-associated genes were screened by ABRicate v1.0.1 against the CARD, ARGannot and ResFinder databases (database datasets updated on 10 October 2022). Plasmid/chromosome contig classification was predicted by RFPlasmid with a posterior probability cutoff value of 0.5. Prokka v.1.14.6 was used for whole-genome functional annotation.

### 2.3. Experimental Animal

The experimental animals used in this experiment were 6-week-old female SPF ICR mice, purchased from the Guangdong Provincial Experimental Animal Center (Guangzhou, China). Animal experiments strictly abide by the principles of experimental animal welfare and ethics. A total of 78 SPF-grade female ICR mice aged 6 weeks with a body weight of 18 ± 1.28 g were used for animal experiments. All mice were purchased from the official Guangdong Provincial Laboratory Animal Center with a stable genetic background and healthy physical status. After transportation to the Laboratory Animal Center of South China Agricultural University, all mice received at least 7 days of quarantine and adaptive feeding. Only mice with stable physiological conditions and confirmed health qualification were included in formal experiments. The laboratory animal use license number is SYXK (Yue) 2019-0136. As a classic experimental animal model, ICR mice have strong environmental adaptability and high sensitivity to microbial stimulation, which is highly suitable for in vivo verification of microbial pathogenicity in food safety research.

In addition, all animal experimental protocols in this study were strictly reviewed and approved by the Institutional Animal Ethics Committee of South China Agricultural University (Approval No. 2022B165, approval date: 27 November 2022). All experimental operations complied with domestic and international laboratory animal welfare guidelines and institutional management specifications. Following the 3R principles (Replacement, Reduction, Refinement), the number of experimental animals and their stress and pain were minimized on the premise of ensuring experimental scientificity. All mice were humanely euthanized according to standard procedures after the experiment, which guaranteed the compliance, scientificity and ethical standardization of this animal study.

For histopathological examination, tissue samples were fixed in 10% neutral-buffered formalin, routinely processed, embedded in paraffin, sectioned at 4 μm thickness, and stained with hematoxylin and eosin (H&E). Sections were examined under a light microscope for pathological changes, including villus atrophy, epithelial necrosis, inflammatory cell infiltration and tissue congestion.

### 2.4. Phylogenetic Analysis

Core genomic single nucleotide polymorphisms (SNPs) were identified using Snippy v4.6.0 (https://github.com/tseemann/snippy accessed on 2 December 202), followed by prediction and filtering of recombination imports using Gubbins v2.4.1 [7]. Maximum likelihood trees were constructed by RAxML (GTRGAMMA alternative model) for filtered core-based SNPs [8]. All phylogenetic trees were annotated with the online tool Interactive Tree of Life (iTOL v6.5.7) and edited for aesthetic purposes.

### 2.5. Determination of Biofilm Production Ability of B. cereus

Biofilm-forming ability was evaluated using the crystal violet semi-quantitative assay. The OD570 value of the eluent was measured with a microplate reader, and biofilm-forming ability was classified according to ODc, the mean OD value of the negative control. Isolates were classified as non-biofilm producers (OD ≤ ODc), weak biofilm producers (ODc < OD ≤ 2ODc), moderate biofilm producers (2ODc < OD ≤ 4ODc) or strong biofilm producers (OD > 4ODc).

### 2.6. Statistical Analysis

All microbiological isolation rates and gene detection frequencies were reported as descriptive percentages. Most-probable-number (MPN) values were calculated strictly following the Chinese national food safety standard GB 4789.14-2014.

The median lethal dose (LD_50_) with corresponding 95% confidence intervals (CIs) for each strain from the mouse acute toxicity assay was calculated by the modified Kärber method using GraphPad Prism 9.0 software. Mortality data from each dosage group (number of animals, number of deaths) were imported for LD_50_ computation.

For biofilm-formation assays, absorbance values at OD_570_ were recorded, and biofilm-producing capacity was classified according to the ODc threshold of negative control wells.

Genomic, MLST and phylogenetic outputs in this study were treated as descriptive observational data; no comparative hypothesis-testing statistical analyses were performed for genomic datasets.

### 2.7. Pathogenicity Test of B. cereus In Vivo

(1) Bacterial strains

Three sausage-origin isolates carrying tigecycline-associated resistance determinants and multiple virulence-associated genes were selected for the mouse acute toxicity assay: BY118LC, TH156LC and TH248-3LC.

(2) Bacterial suspension preparation

Each strain was revived from frozen stock, cultured overnight, washed and adjusted to target concentrations ranging from 1 × 10^4^ CFU/mL to 1 × 10^8^ CFU/mL. Actual viable counts were determined by plate counting immediately before oral gavage.

(3) Acute toxicity test in mice

Four concentrations from 1 × 10^5^ CFU/mL to 1 × 10^8^ CFU/mL were determined by preliminary experiments. A total of 78 6-week-old female ICR mice (18 ± 1.28 g) were randomly divided into 13 groups with 6 mice in each group. After fasting for 24 h, different doses of *B. cereus* were administered. The concentration and number of mice in each group before the experiment are shown in the figure, and the blank group was given the same volume of normal saline. Each mouse was given 0.5 mL by single oral gavage, and clinical signs and mortality were monitored daily for 14 consecutive days thereafter. The mental state and death of the mice were observed at the same time every day (Table 1).

(4) Production of mouse pathological sections

Histopathological sections were prepared using routine histopathological procedures. Briefly, tissue samples were fixed, embedded in paraffin, sectioned and stained with hematoxylin and eosin. Prepared sections were observed under a light microscope, and pathological changes, including epithelial necrosis, villus injury, inflammatory cell infiltration and tissue congestion, were recorded and evaluated.

## 3. Results and Analysis

### 3.1. Isolation Results of B. cereus Group in Milk and Sausage

As shown in Table 2, *B. cereus* group organisms were isolated from 10 of 117 milk samples (8.5%) and 174 of 388 commercially available smoked sausage samples (44.9%). Overall, 184 *B. cereus* group isolates were recovered from the 505 animal-origin food samples. Genome sequencing was subsequently performed on 169 representative isolates for antimicrobial-resistance and virulence-associated gene analysis.

### 3.2. Genome-Wide Analysis Results

#### 3.2.1. Distribution of Drug Resistance Genes

**Figure 1:**
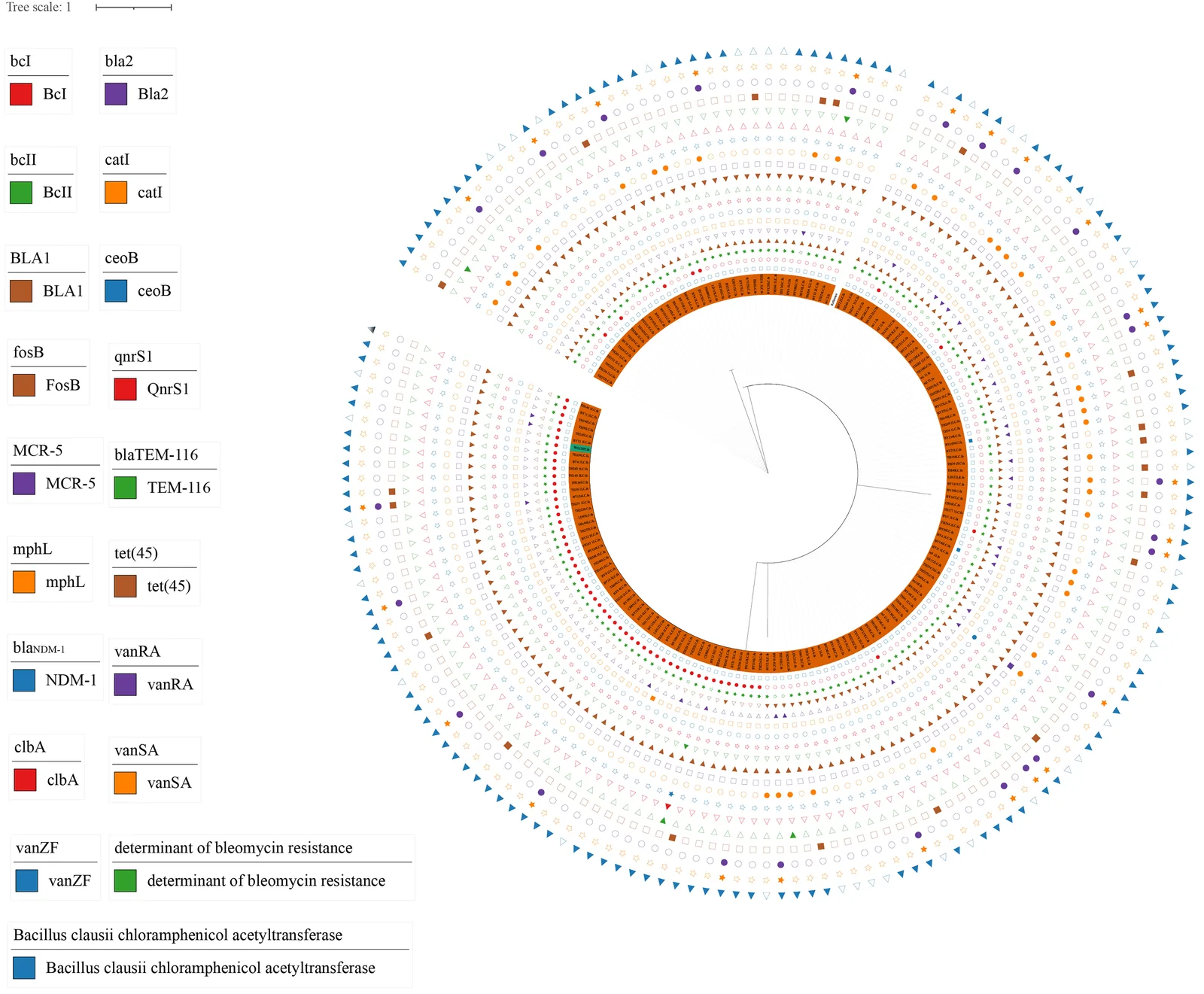
Phylogenetic tree and antimicrobial-resistance gene distribution of 169 sequenced *B. cereus* group isolates.

Figure 1 shows that resistance genes varied significantly across the 169 *B. cereus* strains. The *fosB* gene was by far the most common, present in 99.41% of strains. In contrast, a group of genes including *cat I* and *bla*_NDM-1_ was extremely rare, each occurring in only 0.59% of strains. Other genes such as *bacII* (81.76%) and *BLA1* (65.88%) were relatively common, while genes like *Bla2* (15.88%) and *vanRA/vanSA* (14.71%) were less frequent.

The high prevalence of *bacII*, *fosB* and *vanZF* suggests that genetic determinants linked to intrinsic or putative antimicrobial resistance are widespread at the genomic level among these isolates. In contrast, genes such as *catI*, *ceoB*, *clbA*, the determinant of bleomycin resistance, *mcr-5*, *bla*_NDM-1_ and *qnrS1* were rarely detected. It should be emphasized that genome-based detection indicates the presence of resistance-associated genes but does not alone confirm gene expression, phenotypic resistance or transferability. Therefore, the biological significance of these genes should be interpreted together with antimicrobial susceptibility testing, plasmid localization and expression-level validation.

#### 3.2.2. Distribution of Virulence-Associated Genes

**Figure 2:**
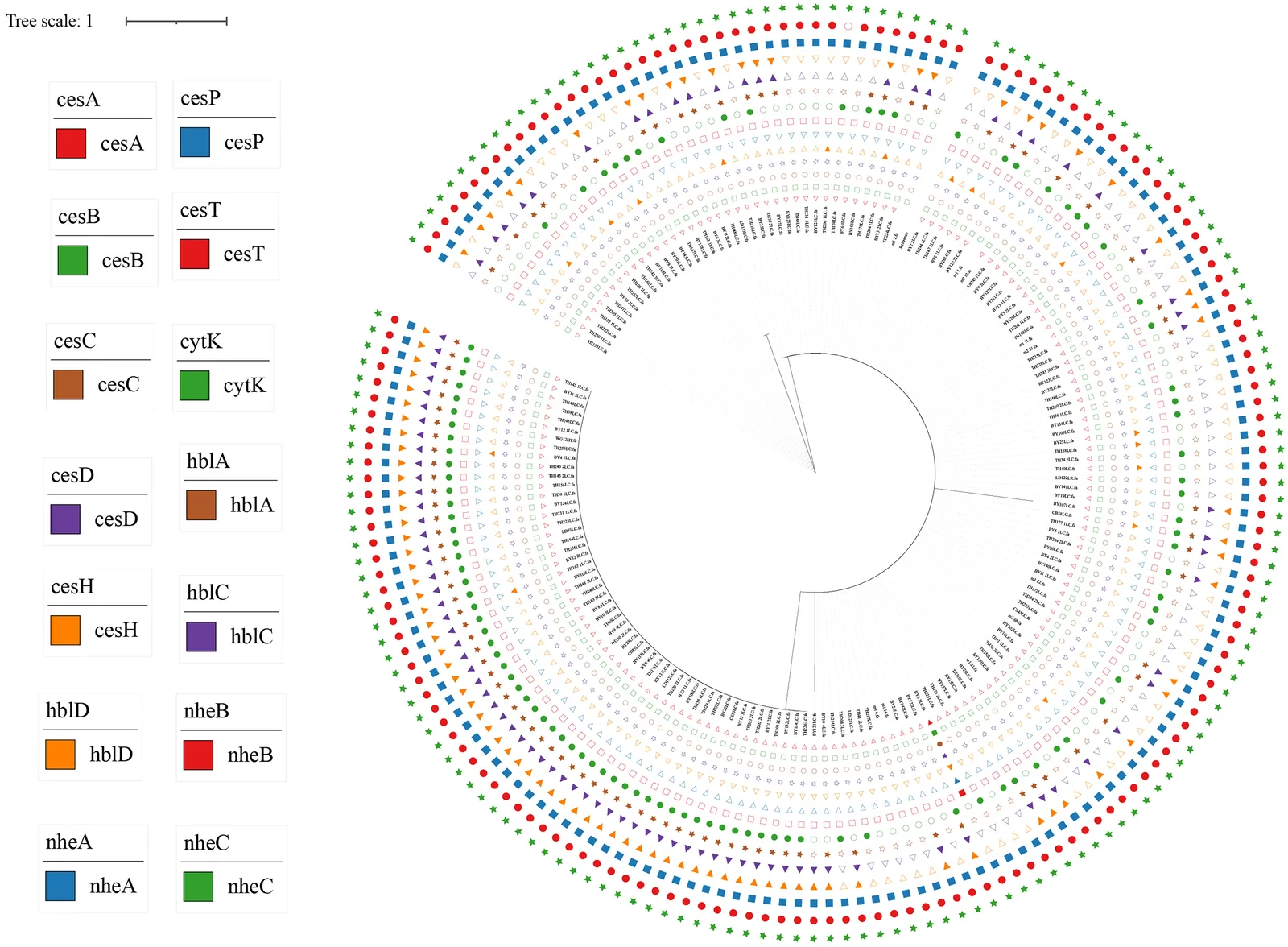
Phylogenetic tree and partial virulence-associated genes distribution of 169 sequenced *B. cereus* group isolates.

Figure 2 reveals a similar variation in virulence-associated genes among the 169 *B. cereus* strains. The *nheA* and *nheC* genes were the most widespread, appearing in all samples (100%), while the *cesA-T* group was extremely rare at only 0.59%. Genes such as *hblC*, *hblD* and *cytK* showed moderate prevalence (around 56~59%), and *nheB* was also very common (99.41%). In comparison, *cesH* had a much lower frequency (10.06%).

The widespread distribution of *nheA*, *nheB* and *nheC* indicates that many isolates carry genomic markers associated with diarrheal pathogenic potential at the genetic level only. The *ces* gene cluster was detected at a low frequency, suggesting that emetic-toxin-associated genotypes were uncommon in this collection. However, the presence of virulence-associated genes does not necessarily indicate active expression, toxin production or clinical disease. Functional assays, toxin quantification and food-matrix challenge studies are still needed to determine the actual pathogenic risk under specific storage and consumption conditions.

### 3.3. Biofilm-Forming Ability of B. cereus

#### Biofilm Production Capacity Measurement Results

**Figure 3:**
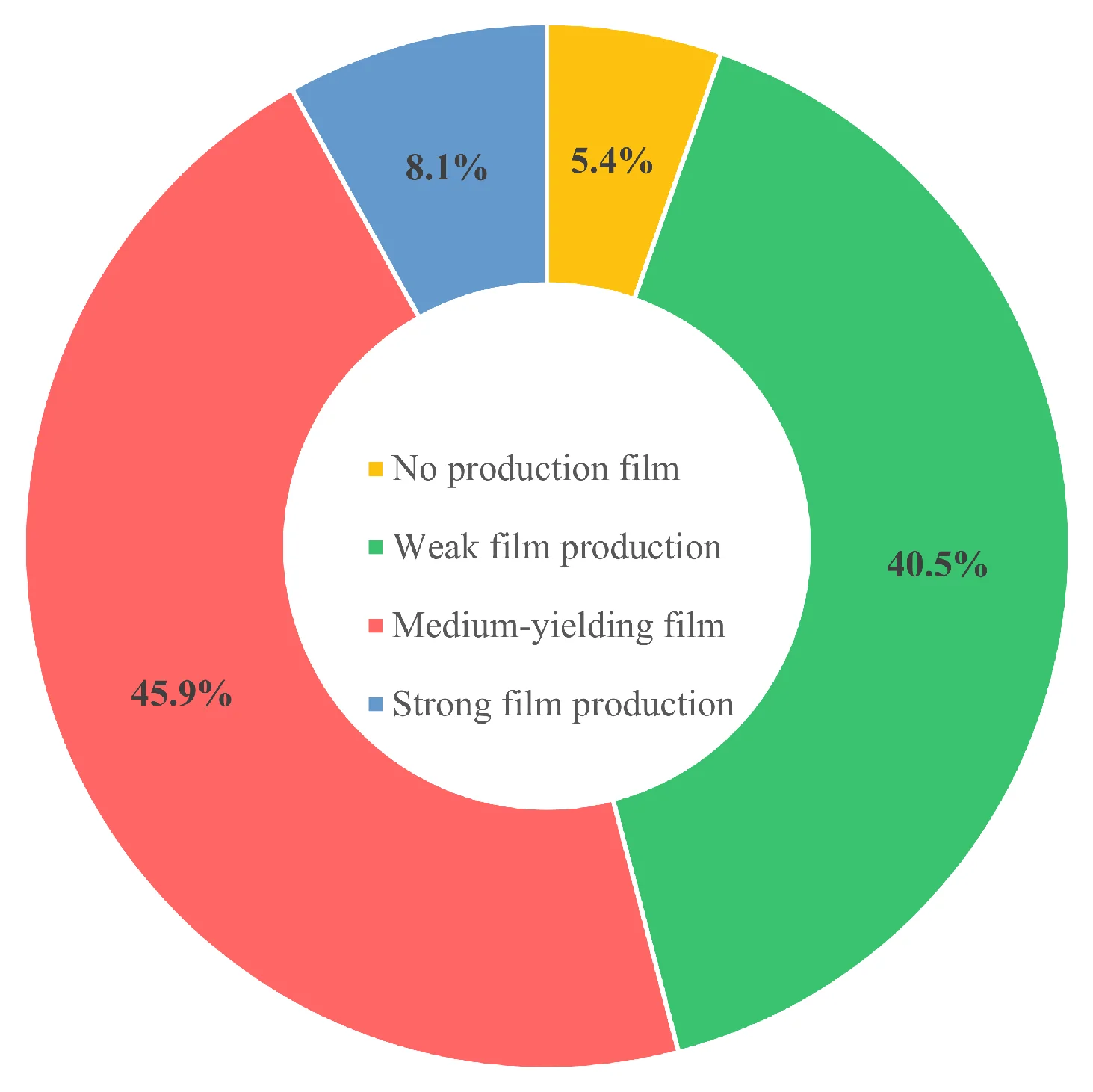
The distribution of biofilm-forming ability of 37 representative isolates.

Using the crystal violet semi-quantitative method, the biofilm-forming ability of 37 representative isolates was measured (Figure 3). Among them, 2 isolates showed no detectable biofilm-forming ability (5.41%), 15 isolates showed weak biofilm-forming ability (40.54%), 17 isolates showed moderate biofilm-forming ability (45.94%), and 3 isolates showed strong biofilm-forming ability (8.11%). According to draft-genome annotation, 37 putative biofilm-associated genes were annotated in 25 isolates, and 20 genes, including members of the pur gene family, *ccpY*, *codY*, *sigB*, *sinR*, *sipW*, *tasA* and *xerC*, were identified in all isolates. Five genes (*epsD*, *epsG*, *epsK*, *epsM* and *epso*) were present in only a small number of isolates, ranging from 5.41% (2/37) to 8.11% (3/37).

### 3.4. Results of Acute Toxicity Test in Mice

Because sausage-origin *B. cereus* group isolates carried multiple enterotoxin-associated genes, three representative isolates (BY118LC, TH156LC and TH248-3LC) carrying tigecycline-associated resistance determinants and multiple virulence-associated genes were selected for the mouse acute toxicity assay to further evaluate their pathogenic potential (Table 3).

### 3.5. Clinical Manifestations in Mice

After infection, mice in the higher dose groups (10^8^ CFU/mL and 10^7^ CFU/mL) of all three strains showed clear signs of illness, including lethargy, reduced appetite, ruffled fur, and diarrhea. Some mice in the 10^7^ CFU/mL group began to recover by the third day. In contrast, those in the low-dose group (10^6^ CFU/mL) only experienced temporary reductions in activity and food intake on the first day, with normal feces and full recovery by the second day. The lowest dose group (10^5^ CFU/mL) and the control group showed no noticeable symptoms throughout the experiment.

Mortality was observed from the second day onward. Deaths in the highest dose group occurred mainly between days 2 and 5, while those in the second dose group occurred between days 3 and 9. After 14 days, all mice in the 10^8^ CFU/mL group of strains BY118LC and TH248-3LC died. In the 10^7^ CFU/mL group, one mouse died for BY118LC and three for TH248-3LC. For TH156LC, five mice died at the 10^8^ CFU/mL dose, but no deaths occurred in lower dose groups. No mortality was observed in any other dose groups across the strains.

### 3.6. LD_50_ of Mice Infected with B. cereus

According to the results of the two-week challenge test, the LD_50_ of different strains was calculated by the modified Karber’s method. As shown in Table 4, the LD_50_ of BY118LC was 2.2 × 10^7^ CFU/mL, the LD_50_ of TH156LC was 9.3 × 10^7^ CFU/mL, and the LD_50_ of TH248-3LC was 3.5 × 10^7^ CFU/mL.

### 3.7. Results of Necropsy and Pathological Section

The dead mice were dissected, and it was found that the intestinal wall of the dead mice became thinner, the intestinal canal was dilated, and the intestinal cavity was filled with a large amount of yellow serous fluid and mucus. The lesions were severe in the large intestine, ileum and duodenum. The liver of the mice was swollen, bleeding and congested, and some of the dead mice had gray–white necrotic spots on the surface of the liver. Occasionally, the spleen was slightly enlarged. No significant gross lesions were observed in other organs. The mice in the blank group injected with the same dose of normal saline were found to have no changes in the organs after autopsy.

**Figure 4:**
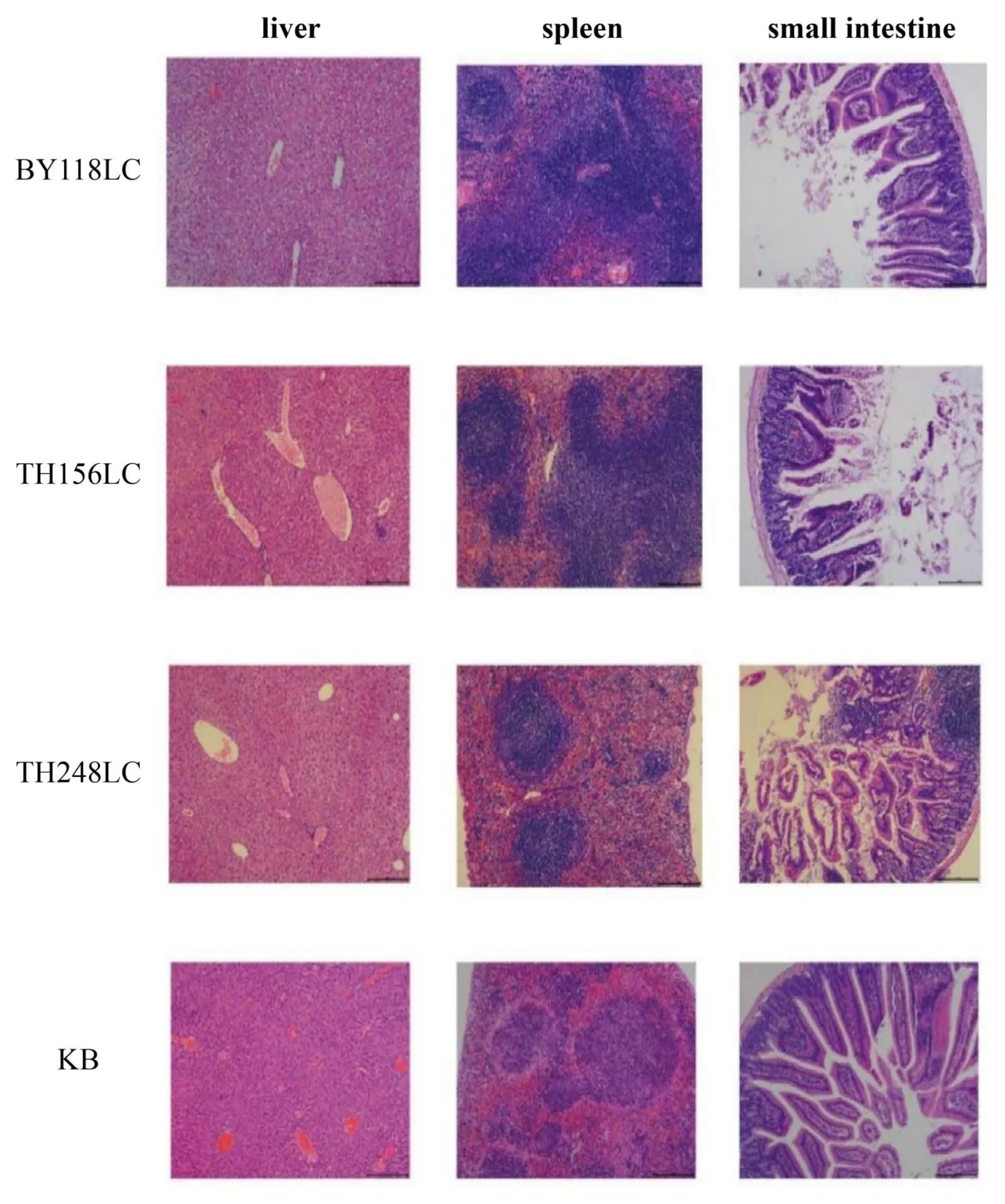
Pathological sections from the acute toxicity test in mice.

Histopathological sections revealed that the liver and small intestine of dead mice after challenge exhibited the most pronounced inflammatory responses, whereas no significant pathological changes were observed in the spleen (Figure 4). Intestinal villi were loosely arranged; epithelial cells in the upper mucosa showed necrosis and exfoliation; exfoliated cell debris was present in the intestinal lumen; and fibrin-like exudates were observed. Inflammatory cell infiltration was observed in the lamina propria, mainly involving lymphocytes, monocytes/macrophages, and a small number of neutrophils and plasma cells. Hepatocytes were swollen with focal necrosis, hepatic sinus narrowing and hemolysis. The red pulp and white pulp of the spleen were clearly demarcated without obvious pathological lesions. These results indicate that the three selected foodborne *B. cereus* group isolates had pathogenic potential in mice under high-dose experimental administration.

## 4. Discussion

### 4.1. Epidemic Characteristics and Genetic Evolution Analysis of B. cereus

Meat and meat products, such as sausages, play an important role in the human diet [9]. Sausages produced via spontaneous fermentation undergo complex biochemical processes involving interactions among various microorganisms, and inadequate quality and safety controls during manufacture make them susceptible to pathogenic microbial contamination [10,11,12]. Animal-origin foods, including fermented and smoked meat products, may be contaminated by *B. cereus* group organisms throughout production, processing, storage and retail. Smoked sausages are complex meat products whose microbiological status is affected by raw material quality, processing conditions, packaging, storage temperature and retail environment. *B. cereus* is one of the most important food-borne pathogens worldwide, and food contamination by this bacterium can cause food poisoning [4]. Therefore, investigating the contamination of *B. cereus* group organisms in such products is of great significance for food safety hazard identification and risk assessment.

In this study, a total of 505 milk and smoked sausage samples were collected from farmers’ markets and large supermarkets in Guangzhou. *B. cereus* group organisms were isolated using traditional isolation, culture and biochemical identification methods, and 184 isolates were obtained. The isolation rates were 8.5% for milk samples and 44.9% for smoked sausage samples, respectively. Among positive samples, 98% contained <1100 MPN/g, while 2% exceeded this threshold; this proportion is close to the 11.87% reported in a 2022 review concerning *B. cereus* contamination [13]. Kong et al. investigated *B. cereus* contamination in 603 meat and meat product samples collected from 39 cities across China, reporting an overall contamination rate of 27.03%, with 4.9% of positive samples exceeding 1100 MPN/g [14]. According to Fouad’s findings, the high prevalence of *B. cereus* group organisms in sausages is closely associated with the quality of raw starting materials [15]. Moreover, most sausages are not subjected to heat treatment during manufacturing and lack refrigeration, and such production and storage practices substantially increase the risk of microbial contamination [16]. In addition, many commercially available sausages are sold in bulk and exposed to ambient air without packaging protection, allowing contact with spore-containing dust and thereby facilitating contamination by *B. cereus* group organisms [17]. However, isolation of *B. cereus* group organisms from a food product should not be interpreted as direct evidence that the organism multiplied within the food matrix or that consumers are exposed to a hazardous dose. In this study, the concentrations detected in 98% of positive samples were <1100 MPN/g, complying with the limits specified in GB 29921-2021 (Chinese National Food Safety Standard for Contaminants in Food) for relevant prepackaged foods. These results indicate a potential microbiological hazard that warrants attention, but they do not constitute a quantified consumer-risk assessment.

### 4.2. Virulence Potential Characterization, Virulence Gene Profiling and Consumer Risk Assessment of B. cereus Group

Yi et al. collected milk samples from a dairy farm in Chongqing and isolated a strain of *B. cereus*. PCR results showed that the strain carried virulence-associated genes including *hblA*, *hblC*, *hblD*, *nheA*, *nheB*, *nheC*, *cytK* and *entFM*. The mouse toxicity test showed that the isolate had an LD_50_ of 1.44 × 10^10^ CFU in mice and exhibited significant hepatotoxicity in mice [18]. In 2021, China promulgated the National Food Safety Standard Limits for Pathogenic Bacteria in Bulk Instant Foods. The document stipulates that the content of *B. cereus* in foods made with rice as the main raw material must be less than 10^4^ CFU/g (mL). Studies have demonstrated that spores of pathogenic *B. cereus* can germinate and multiply under suitable storage temperature, storage duration or cooking treatments after food contamination. When bacterial loads exceed 10^5^ CFU/mL, emetic or diarrheal toxins are readily produced, thereby triggering emetic or diarrheal foodborne illnesses [19]. Wang Jing et al. performed a risk assessment of harmful microorganisms in fermented meat products. Their results indicated that sales temperature, storage temperature and storage time were major risk factors driving the proliferation of *B. cereus*, and frozen storage throughout shelf-life determined the final concentration of *B. cereus* in food products [20]. These observations highlight the necessity to control contamination and storage conditions during sausage production and retail sale.

The detection of virulence-associated genes is useful for evaluating pathogenic potential, but gene presence alone cannot prove transcription, toxin production or disease risk. Similarly, detection of antimicrobial-resistance-associated genes does not necessarily confirm phenotypic resistance, gene expression or gene transferability. Therefore, genomic data should be interpreted together with phenotypic antimicrobial susceptibility testing, toxin quantification, biofilm assays, plasmid localization and food-matrix challenge studies.

Whether *B. cereus* group organisms can multiply in sausage depends on product formulation and storage conditions. *B. cereus* group spores may survive some processing procedures; germination and vegetative growth are jointly regulated by temperature, pH, water activity (aw), salt concentration, preservatives, competing microbiota, packaging and storage time. Some *B. cereus* group strains can grow at temperatures as low as 5 °C, and the approximate lower growth limits across this group are pH 5.0 and aw 0.93 [21]. Consequently, fresh, cooked, or insufficiently acidified/dried sausages under poor time-temperature control may support bacterial proliferation, whereas validated combinations of heating, acidification, drying, preservatives and refrigeration restrict bacterial growth. A recent survey in southern China recovered *B. cereus* group organisms from 19.1% (60/314) of sausage samples; sausage isolates exhibited the highest species diversity among all tested foods, and many sequenced isolates carried diarrheal toxin genes [5]. These findings, together with our 44.9% isolation rate and virulence-associated traits of our isolates, suggest a potential microbiological hazard. Nevertheless, the present study did not directly demonstrate growth of *B. cereus* group organisms in sausage matrices or quantify consumer exposure at consumption. In our study, 98% of positive samples contained <1100 MPN/g, and the detected loads were within the limits specified in GB 29921-2021 (National Food Safety Standard—Limits of Pathogens in Prepackaged Foods) [22]; no samples exceeded the stipulated thresholds, while most reported *B. cereus* group outbreaks have been associated with concentrations >10^5^ CFU/g [21]. Product-specific challenge tests measuring *B. cereus* populations together with pH, water activity and storage conditions are required to determine whether particular sausage products support bacterial growth and reach hazardous levels.

Notably, TH156LC and TH248-3LC shared an identical set of 25 virulence-associated genes and clustered adjacently in phylogenomic trees (Figure 1 and Figure 2), indicating a potential close genetic relationship. However, high-resolution SNP typing is required to confirm their clonality. A biofilm is a complex structure formed by aggregated bacterial cells, which protects bacteria against environmental stressors and external threats such as antibiotics [23]. Many pathogenic bacteria possess strong biofilm-forming capacity, which enables them to evade immune clearance and antibiotic killing [24]. In addition, biofilms facilitate bacterial colonization and proliferation within hosts and consequently enhance pathogenicity [25]. Therefore, biofilm-forming ability is generally recognized as an important contributor to bacterial virulence. In this study, genome annotation identified 25 biofilm-associated genes among 37 *B. cereus* group strains. Crystal violet semi-quantitative assays showed that 94.6% of the isolates were capable of biofilm formation. Analysis of virulence-associated genes is an important approach to explore the pathogenic mechanisms of the *B. cereus* group. To date, multiple virulence-associated genes related to the pathogenicity of the *B. cereus* group have been identified. The presence of virulence-associated genes serves as an important indicator for evaluating the pathogenic potential of isolates, and multiple detection methods are continuously being optimized. However, several issues remain to be further investigated. For instance, the interactions among virulence-associated genes and their synergistic mechanisms with environmental factors are not fully elucidated; functional studies on newly discovered virulence genes or gene mutations need to be strengthened; and rapid, simple and high-throughput detection methods for *B. cereus* virulence-associated genes still require further development. Nevertheless, the relationship between biofilm-associated genes, biofilm phenotype, virulence-gene expression and actual bacterial growth in sausage matrices requires further functional validation.

## 5. Conclusions

This study shows that *B. cereus* group organisms were frequently recovered from animal-origin foods sold in Guangzhou. The isolation rates were 8.5% (10/117) in milk samples and 44.9% (174/388) in commercially available smoked sausage products. Whole-genome sequencing of 169 representative isolates showed that many sausage-derived isolates harbored antimicrobial-resistance-associated and virulence-associated genes, and most representative isolates possessed biofilm-forming ability. Mouse experiments further revealed that selected isolates carrying multiple antimicrobial-resistance and virulence-associated genes exhibited pathogenic effects under artificial high-dose experimental administration in laboratory animals. Nevertheless, it should be emphasized that milk samples were only subjected to isolation-rate investigation; whole-genome sequencing, biofilm phenotypic assays and mouse pathogenicity experiments were performed exclusively on sausage-origin isolates. Therefore, the detection of *B. cereus* group organisms and virulence-associated genes should be interpreted as evidence of a potential microbiological hazard rather than direct proof of consumer risk. This study did not evaluate growth kinetics of *B. cereus* group in sausage matrices, toxin production during storage or actual exposure levels at consumption. Moreover, the high-dose mouse challenge model does not directly represent normal dietary exposure. Future product-specific challenge studies should evaluate *B. cereus* group growth, pH, water activity, storage temperature and storage time in smoked sausage products to support quantitative risk assessment and targeted food-safety control.

## Figures and Tables

**Table 1:** Experimental grouping and attack dose.

Strain	Infection Dosage (CFU/mL)				
	The First Group	The Second Group	The Third Group	The Fourth Group	Blank
BY118LC	10^5^	10^6^	10^7^	10^8^	0.9% sterilized saline water
TH156LC	10^5^	10^6^	10^7^	10^8^	
TH248-3LC	10^5^	10^6^	10^7^	10^8^	

Note: nominal concentrations indicate target concentrations used during bacterial suspension preparation. Actual viable bacterial concentrations were determined by plate counting immediately before gavage. Minor deviations between nominal and actual values are inherent to bacterial dilution procedures.

**Table 2:** Statistical table on isolation of *B. cereus* group from milk and sausage in supermarkets and farmers’ markets in Guangzhou.

Source	No. of Samples	No. of Positive Samples	Isolation Rate (%)
Milk	117	10	8.5
Sausage	388	174	44.9

**Table 3:** The virulence-associated genes carried by the three test strains.

Strain	Enterotoxin Virulence-Associated Genes Carried	Quantity/Unit
BY118LC	*entFM*, *plcA*, *cerA*, *nheA*, *hblD*, *sph*, *bceT*, *inhA2*, *cytK2*, *clo*, *entA*, *plcR*, *nheC*, *hblC*, *nheB*, *bpsE*, *bpsF*, *plcB*, *hblB*, *inhA1*, *cerB*, *hblA*, *bpsH*, *hlyII*, *hlyR*	25
TH156LC	*entFM*, *bpsD*, *plcA*, *cerA*, *nheA*, *hblD*, *sph*, *bceT*, *inhA2*, *cytK2*, *clo*, *entA*, *plcR*, *nheC*, *hblC*, *nheB*, *bpsE*, *bpsF*, *plcB*, *hblB*, *inhA1*, *cerB*, *hblA*, *bpsH*, *hlyR*	25
TH248-3LC	*entFM*, *bpsD*, *plcA*, *cerA*, *nheA*, *hblD*, *sph*, *bceT*, *inhA2*, *cytK2*, *clo*, *entA*, *plcR*, *nheC*, *hblC*, *nheB*, *bpsE*, *bpsF*, *plcB*, *hblB*, *inhA1*, *cerB*, *hblA*, *bpsH*, *hlyR*	25

**Table 4:** Median lethal dose of *B. cereus*.

Strain	Gavage Dosage (CFU/mL)	Size of Animal (Piece)	Dead Number (Piece)	Mortality (%)	Median Lethal Dose (CFU/mL)
BY118LC	1 × 10^8^	6	6	100	2.2 × 10^7^
	1 × 10^7^	6	1	16.7	
	1 × 10^6^	6	0	0	
	1 × 10^5^	6	0	0	
TH156LC	2 × 10^8^	6	5	83.3	9.3 × 10^7^
	2 × 10^7^	6	0	0	
	2 × 10^6^	6	0	0	
	2 × 10^5^	6	0	0	
TH248-3LC	3.5 × 10^8^	6	6	100	3.5 × 10^7^
	3.5 × 10^7^	6	3	50	
	3.5 × 10^6^	6	0	0	
	3.5 × 10^5^	6	0	0	
Normal saline	500 μL	6	0	0	/

Note: Actual gavage dosage is expressed as CFU per gram of body weight, calculated from the measured viable bacterial concentration, gavage volume (0.5 mL), and average mouse body weight (~18 g). All LD_50_ values were calculated using the modified Kärber method based on actual measured viable counts, not on nominal concentrations.

## Data Availability

The original contributions presented in this study are included in the article. Further inquiries can be directed to the corresponding authors.
